# COVID-19 pandemic containment in the Caribbean Region: A review of case-management and public health strategies

**DOI:** 10.3934/publichealth.2021053

**Published:** 2021-09-27

**Authors:** Srikanth Umakanthan, Anuradha Chauhan, Madan Mohan Gupta, Pradeep Kumar Sahu, Maryann M Bukelo, Vijay Kumar Chattu

**Affiliations:** 1 Department of Para-clinical sciences, Faculty of Medical Sciences, St Augustine, The University of the West Indies, Trinidad & Tobago, WI; 2 Department of Psychology, University of Toronto Scarborough Campus, Toronto, ON M1C 1A4, Canada; 3 School of Pharmacy, Faculty of Medical Sciences, The University of the West Indies, St. Augustine, Trinidad & Tobago, WI; 4 Centre for Medical Sciences Education, The University of the West Indies, St. Augustine, Trinidad & Tobago, WI; 5 Department of Anatomical Pathology, Eric Williams Medical Sciences Complex, North Central Regional Health Authority, Trinidad and Tobago, West Indies; 6 Division of Occupational Medicine, Department of Medicine, Faculty of Medicine, University of Toronto, Toronto, ON, M5S 1A8, Canada; 7 Department of Public Health, Saveetha Medical College and Hospitals, Saveetha Institute of Medical and Technical Sciences, Saveetha University, Chennai-600077, India; 8 Institute of International Relations, The University of the West Indies, St. Augustine, Trinidad and Tobago, WI

**Keywords:** COVID-19, Caribbean, pandemic, transmission, quarantine, vaccine

## Abstract

COVID-19 emerged initially from Wuhan, Hubei province, China, in late December 2019, and since then, it has spread globally to be declared a pandemic by the World Health Organization. The Caribbean region started reporting COVID-19 cases in early March 2020, triggering new regional public health crises. The initial suspects and confirmed cases across the Caribbean countries were mainly imported cases and from cruise ships. The clinical manifestations varied from fever, cough, and malaise in mild cases to acute respiratory distress syndrome (ARDS) and shock in severe cases. The Caribbean Public Health Agency has provided frequent updates on the preventive strategies and quarantine measures across the Caribbean member states. COVID-19 has had a serious impact on the Caribbean region's health system, economy, and psychology. This review presents the Caribbean perspective of COVID-19, detailing the epidemiology, clinical manifestations, diagnosis, management, and preventive and surveillance measures. Vaccine hesitancy was found to be a major challenge that needs appropriate health education strategies to address the public. Strong leadership and regional collaboration among the Caribbean member states are necessary to provide optimal real-time data to the public and implement appropriate and effective guidelines in the island states.

## 1. Introduction

The severe acute respiratory syndrome coronavirus 2 (SARS-CoV-2) was identified as the cause of the current COVID-19 pandemic that began in December 2019 in Wuhan city of China. Historically viral diseases have been causing a disastrous effect on global health and the economy [1]–[3]. Based on global statistics, as of Jul 7, 2021, around 185 million confirmed cases and 4 million reported deaths due to COVID-19 [4]–[6]. The first case in the Caribbean region was reported on Mar 10, 2020, in Jamaica, and as of Jul 7, 2021, 957,946 confirmed cases and 12,877 deaths had been reported in the entire region. Diagnosis is confirmed by a specific molecular method known as real-time polymerase chain reaction (RT-PCR). Management is dependent on the patient's disease state and ranges from observation in mild cases to mechanical ventilation in severe cases [2]. Coronavirus is an RNA enveloped virus composed of five genomic proteins, namely spike protein (S), membrane protein (M), nucleocapsid protein (N), an envelope protein (E), and the hemagglutinin-esterase (HE) protein. The spike (S) protein on its surface gives it a “crown” like appearance, hence the name “corona” [7]. In humans, OC43, NL63, HKU1, and 229E have caused mild respiratory illness. SARS and MERS have been the reason for severe respiratory diseases in the past [8]. SARS was first recognized in Guangdong province, China, in November 2002 and advanced to 30 countries, infecting 79,000 people by 2003 with a fatality rate of 9.5%. SARS-CoV was traced and isolated from Himalayan palm civets found in the livestock market in Guangdong, China [9]. MERS-CoV was initially identified in 2012, Jeddah, Saudi Arabia, in a patient with pneumonia and features of renal failure. The patient's sputum analysis was done by RT-PCR, revealing the viral RNA to be MERS-CoV which infected 91 patients and had a high fatality rate of 34%. Bats and Arabian dromedary camels were identified as potential hosts for MERS-CoV [10].

In late December 2019, a cluster of patients presented to local hospitals in Wuhan, Hubei province of China, with fever, cough, and malaise. These patients had a common exposure to wild animals and were involved in the live animal trade in Huanan wholesale seafood market. A diagnosis of severe atypical pneumonia of unknown etiology was established and subsequently notified to the World Health Organization (WHO) [1]. The Chinese government closed and sanitized the Huanan seafood market, followed by contact tracing for suspect cases. On Jan 7, 2020, the suspect virus was identified as novel CoV-2019. The next few weeks experienced an exponential increase in the number of patients presenting with similar clinical manifestations. Wuhan has been a major transportation hub caused an epidemic to spread to other countries such as Thailand, South Korea, Singapore, and Japan. Travel restrictions, quarantine, and isolation of suspect cases were quickly initiated in many countries [11]. COVID-19 has an epidemic doubling time of 1.8 days forcing the WHO to announce COVID-19 as a global pandemic and provide frequent updates on disease statistics, case definitions, and preventive strategies [5]. In terms of COVID-19 transmission dynamics, a study by Coccia et al. found that cities with high atmospheric stability, as measured by low wind speed, and frequently high levels of air pollution – exceeding safe levels of ozone or particulate matter – had a higher number of COVID-19-related infected individuals and deaths [12]. The authors further documented that transmission dynamics of COVID-19 is due to air pollution-to-human transmission rather than human-to-human transmission [13]. A systematic review by Chattu et al. has highlighted that to combat the COVID-19 epidemic, the focus areas must be on equity, health promotion, and sustainable development [14]. A recent study found that the best-performing countries in dealing with the COVID-19 pandemic issue have a smaller population and/or stronger public governance, as well as high healthcare spending [15].

If SARS-CoV-2 gets entrenched in the community, COVID-19 vaccines will be critical in the future for lowering morbidity and death and generating herd immunity [16]. However, even if vaccine supplies were available, the US public health system was unprepared and understaffed to handle the duty of delivering >450 million doses of vaccine in a timely manner. Failure to invest sufficiently in public health has been prevalent across the country at all levels of government [17].

In the Caribbean region, vaccine hesitancy is a significant impediment to vaccine uptake and herd immunity, which is essential to protect the most susceptible populations. Therefore, the educational campaigns also should include information about the contribution of an individual's immunization to herd immunity. Besides, transparency about vaccine effectiveness and adverse events to set public expectations will likely improve trust in a COVID-19 vaccine, but messaging should take care to avoid unintentional overemphasis on the risk of rare adverse events [18]. In the United States, in the initial phase of the immunization campaign, the Advisory Committee on Immunization Practices (ACIP) advised that both 1) health care professionals and 2) residents of long-term care institutions be administered the COVID-19 vaccine [19]. Using nationally representative data from general adult populations in Ireland and the United Kingdom, the vaccine hesitancy/resistance was 35% and 31%, respectively [20].

Similarly, a French study found a vaccine hesitancy among the working-age population ranged from 9.3% to 43.2%, depending on the vaccine characteristics [21]. Besides, from a developing country perspective, a study from Uganda on the acceptance of the COVID-19 vaccine found a low level of interest for vaccine and clinical trial interest [22]. In the United States, Viswanath et al. have concluded in their study that Race/ethnicity, risk perceptions, exposure to diverse media for COVID-19 news, party identity, and trust in scientists were found as variables influencing COVID-19 vaccination uptake [23]. The vaccine hesitancy in the Caribbean region is also found to be high, and furthermore, the region has suffered a lot because of the impact of the pandemic due to its geographical, financial, and technological limitations. In this context, we aimed to present the containment of COVID-19 from the Caribbean perspective with the following objectives: 1) to discuss the epidemiology (including transmission dynamics), clinical manifestations, diagnosis, and case-management, and 2) to describe the surveillance systems, various preventive measures and vaccination plan including vaccine hesitancy of the population.

## 2. Materials and methods

A literature search was performed in MEDLINE/PubMed. We used PubMed's MeSH terms COVID-19, global statistics, Caribbean, pandemic, transmission, quarantine, vaccine, and surveillance. Abstracts were screened, focusing on the search results in relevance to this review. Cochrane reviews, systematic reviews, and meta-analyses were prioritized. Articles focusing on COVID-19, global and Caribbean statistics, and management strategies were prioritized. This review search included COVID-19 relevant articles, references, keywords, and the ‘similar article’ tab on the PubMed database. This approach generated 72 relevant research articles, including 32 reviews, 29 original articles, 11 website searches, and one case report published from 2003 to 2021. During the search, contradictory views, results, and subjective conclusions were thoroughly evaluated and referred to relevant source studies before being presented. To access the COVID-19 data, we have referred to the websites of the Caribbean Public Health Agency (CARPHA), Caribbean Tourism, and WHO Dashboard for COVID-19. The region-specific and country-specific data on infections and deaths were collected and shown in graphs and tables. The main findings, such as the burden of the COVID-19 in the region, the case management, the vaccination and vaccine hesitancy, and public health surveillance aspects, are discussed below.

## 3. Results

**Figure 1.**
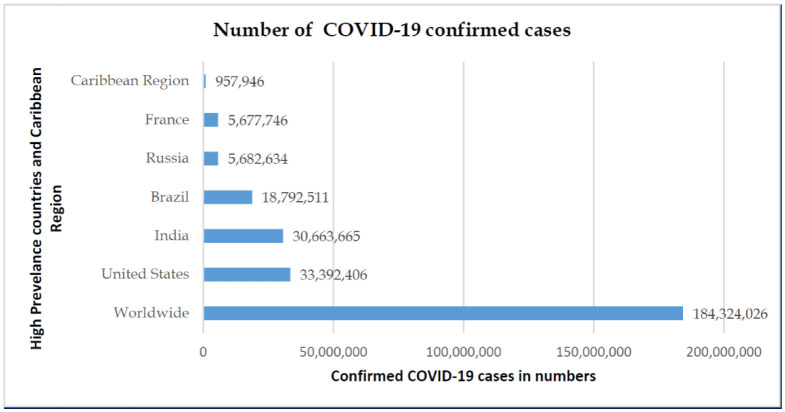
Global statistics and comparisons of confirmed COVID-19 cases.

**Figure 2.**
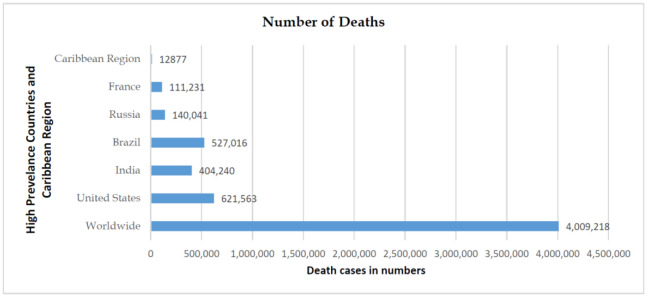
Global statistics and comparison of COVID-19 deaths.

**Figure 3.**
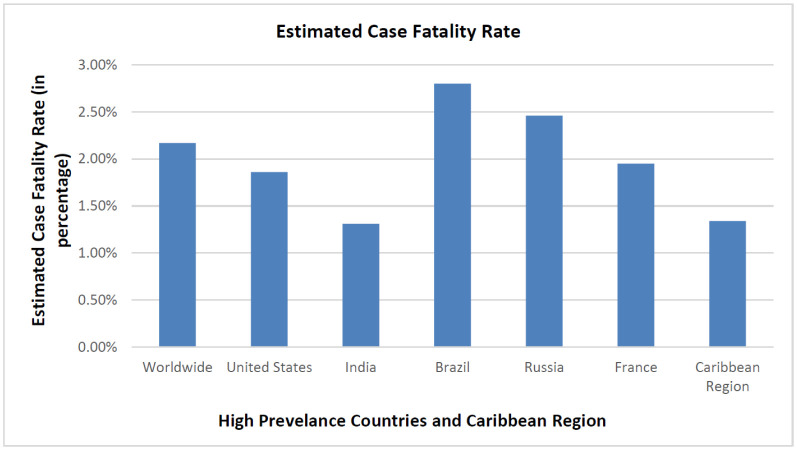
Global statistics and comparison of COVID-19 case fatality rate.

The health and financial impact of COVID-19 on a region are dependent on the epidemic curve, duration of the pandemic, the regional population, government, and health authority's measure on disease containment. Based on July 1, 2021, WHO regional situation reports, United States has the highest number of infected cases, followed by India, Brazil, Russia, and France (Figure 1). The case fatality rate (CFR) was highest in Brazil at 2.80% [5]. The Caribbean region's CFR is at a lower percentage (1.34%.) in comparison to the worldwide CFR (2.17%) (Figures 2 and 3).

**Figure 4.**
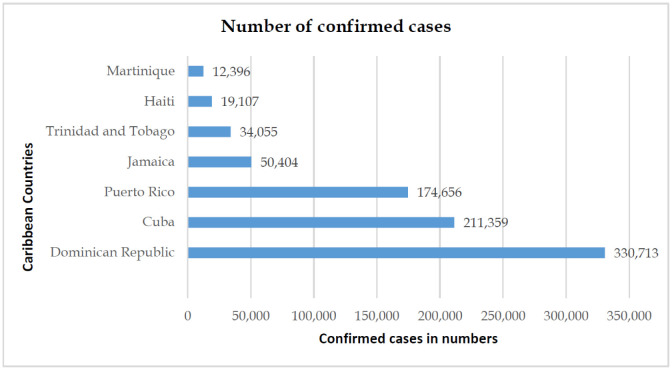
Caribbean region confirmed COVID-19 cases.

The Caribbean region has around 700 islands with approximately 2,25,996 km^2^ of total area with a population of 43,507,721 [24]. The Caribbean region was hit with its first COVID-19 case on Mar 10, 2020, in Jamaica. This was followed by a rapid spread across the region with noticeable spikes on Mar 21 (55 new cases arrived in Trinidad and Tobago from cruise ship), Mar 26 (33 confirmed cases), and Mar 29 (32 confirmed cases). As of July 7, 2021, there have been 957,946 confirmed cases and 12,877 deaths in the Caribbean region. The region-wise statistical analysis (Figure 4) shows the Dominican Republic constitutes 42.53% of confirmed cases in the Caribbean region, followed by Cuba (13.51%) and Puerto Rico (10.48%). These northern Caribbean islands are close to the United States and have frequent flight access to European regions, causing a higher percentage of confirmed imported cases. The confirmed cases in Southern Caribbean islands were from positive cases arriving from cruise ships followed by limited cases of local transmission [2],[25].

**Figure 5.**
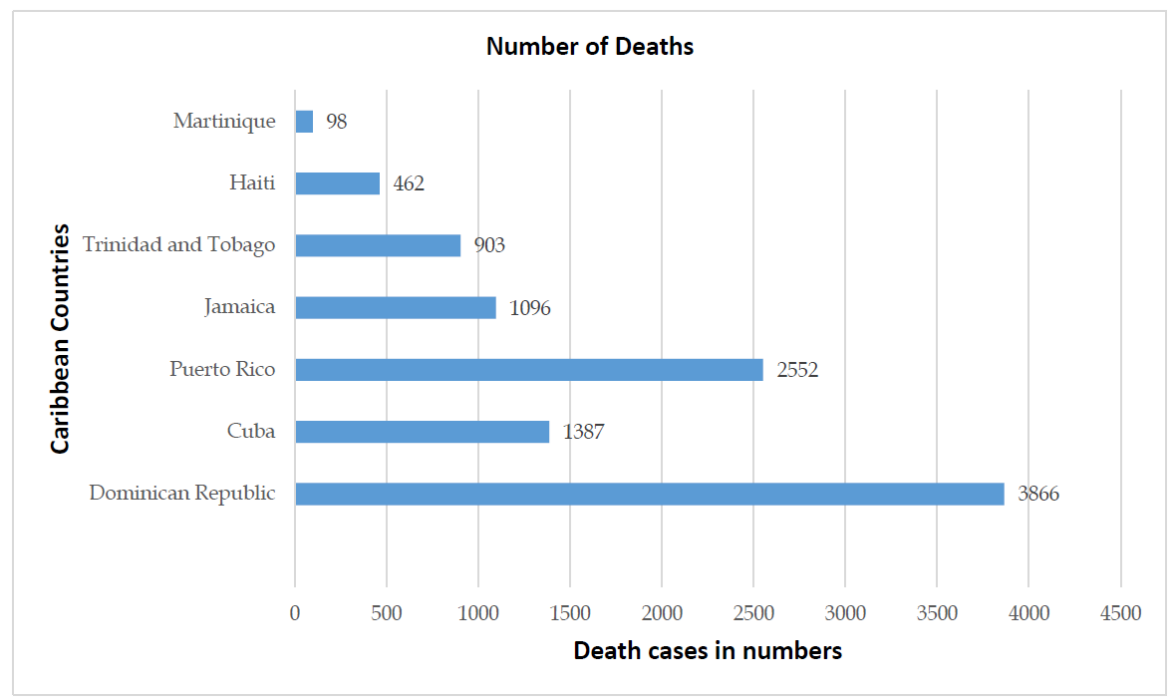
Caribbean region deaths due to COVID-19.

**Figure 6.**
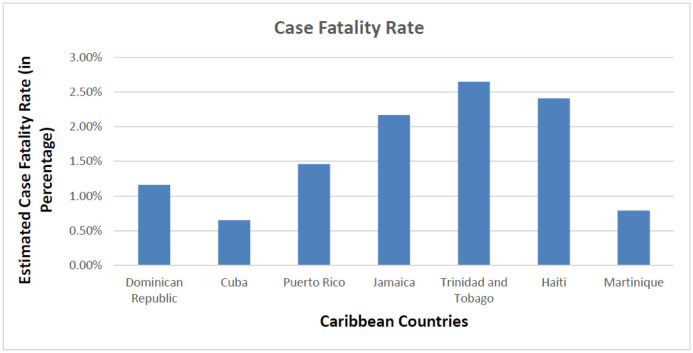
The case fatality rate in the Caribbean region due to COVID-19.

The Dominican Republic and Cuba have managed to keep the CFR (Figures 5 and 6) at the lowest among its member states, owing mainly to its positive health factors. These countries have free healthcare services and also poses a high doctor-to-population ratio. The region's national emergency structure is diverse and linked closely with local organizations enabling easier contact tracing, immediate testing of suspect cases at regional health centers, and faster hospital admissions and case management. These factors have positively impacted COVID-19 patient management and reduced CFR due to COVID-19 complications [26],[27].

At the other end of the spectrum is the CFR in Haiti (Figure 6), standing at an alarmingly high rate of 2.41% compared to its other Caribbean countries. Based on the Human Development Index, Haiti is ranked 169 out of 189 countries. This financial crisis, along with ongoing political crises, frequent episodes of natural calamities, and an ill-equipped health care system, has caused a delirious effect on the management of the present COVID-19 pandemic [28].

## 4. Discussion

Caribbean demographics based on CARPHA statistics show the median age of COVID-19 patients was 50 years (modal range 55–59 years), and women were predominately affected, unlike the global male preponderance for COVID-19. The median onset of illness was 8 days (range 5 to 13 days) [24]. Due to limited comorbid data availability, it is important to correlate with previously proven susceptible factors to SARS and MERS-CoV infection, including smoking, hypertension, diabetes, cardiovascular disease, and/or chronic illness. The striking target to the elderly population is attributed to underlying chronic disorders and declined immune function [29]. Declined immune function has been linked to cytokine storm syndrome (elevated circulating inflammatory cytokines) and hyper-inflammation syndrome. These syndromes are triggered by viral infections and are also predictors of fatality in COVID-19 patients [30]. Children in the Caribbean were less affected due to higher antibodies, lower prior exposure to the virus, and relatively low levels of inflammatory cytokines in their systems [31].

Extensive structural analyses revealed atomic-level interactions between the coronavirus and the host. Cross-species and human-to-human transmission of COVID-19 is mainly dependent on the spike protein receptor-binding domain (RBD) and its host receptor angiotensin-converting enzyme-2 (ACE2). High expression of ACE2 was identified in the lung (type II alveolar cells), esophagus, ileum, colon, kidney (proximal convoluted tubules), myocardium, bladder (urothelial cells), and also recently the oral mucosa. ACE2 receptors provide entry of the virus into the host cells and subsequent viral replication. The main factors involved in viral pathogenesis of 2019-nCov are spike 1 subunit protein, priming by transmembrane protease serine-2 (essential for entry and viral replication), ACE2 receptor-2019-nCov interaction, and downregulation of ACE2 protein. These factors contribute to atrophy, fibrosis, inflammation, and vasoconstriction resulting in host tissue injury [32].

*Modes of transmission:* The Caribbean region is famous for its cultural heterogeneity, diverse ecosystem, and highly demanding tourist beach resorts and cruise voyages. The initial cases across the island countries were mainly imported cases. These patients had flown down to their homeland after visiting either European countries or the United States [3]. This was followed by human-to-human transmission occurring through common routes such as direct, contact, and airborne transmissions through aerosols and medical procedures. Infected cases based on the transmission mode constituted 28% imported cases, 53% local transmission, 58% sporadic cases, and 42% cluster transmission [25]. Cough, sneeze, droplet inhalation, contact with oral, nasal, and eye mucous membranes are the common modes of spread. Viral shedding occurs from the respiratory tract, saliva, feces, and urine, resulting in other sources of virus spread. The viral load is higher and of longer duration in severe COVID-19 patients. The spread of COVID-19 from patients to health workers and flight attendees who were in close contact with the infected patients was also reported [33]. An Italian study by Coccia et al. suggests that high concentrations of air pollutants, associated with low wind speeds, may promote a longer permanence of viral particles in the polluted air of cities, thus favoring an indirect means of diffusion of COVID-19 [34]. Cities with more than 100 days per year exceeding PM_10_ or ozone restrictions, cities located in hinterland zones (i.e., distant from the coast), cities with a low average wind speed, and cities with a lower average temperature had a greater number of COVID-19 infections. Furthermore, the first wave of the COVID-19 pandemic in Italy resulted in more than 75% of infected persons and 81% of fatalities in industrialized districts with high levels of air pollution [35].

*Clinical features and diagnosis:* COVID-19 manifests with varied signs and symptoms depending on the host viral load and virus-host interaction. The most common symptoms include fever, cough, and malaise. Other less reported features include headache, vomiting, diarrhea, ageusia, and conjunctivitis [36]. The COVID-19 was clinically classified into: mild to moderate disease (non-pneumonia and pneumonia), severe disease (dyspnea, respiratory frequency over 30/min, oxygen saturation less than 93%, PaO2/FiO2 ratio less than 300, and/or lung infiltrates more than 50% of the lung field within 24–48 hours) and critical (respiratory failure, septic shock, and/or multi-organ dysfunction/failure) [37]. The progress of COVID-19 from mild illness to severe form depends on the circulatory levels of inflammatory cytokines and chemokines such as IL-7, IL-8, IL-9, IL-10, GCSF, GMCSF, TNFα, and VEGFA [38]. Complications include severe pneumonia, acute lung injury, ARDS (Acute Respiratory Distress Syndrome), shock, and multi-organ failure [1]. Suspected COVID-19 case presenting with or without symptoms requires molecular laboratory confirmation using RT-PCR test. RT-PCR tests are preferred since they have dual analysis and sequencing. Specimen samples can be obtained from the respiratory passages (Nasopharyngeal swabs, anterior nares, and sputum), oropharyngeal swabs, rectal swabs, fecal specimens, and urine. Suppose the initial RT-PCR test result is negative in a strongly suspected case of COVID-19. In that case, a repeat specimen collection is required from deeper and higher viral load sites such as tracheal aspirates, broncho-alveolar lavage, pleural fluid, and in extreme cases, a lung biopsy would be warranted. The negative result can be attributed to technical errors, improper specimen collection, storage, and transport [39],[40].

Laboratory findings vary from leukocytosis, leukopenia, lymphopenia, elevated AST, reduced albumin, elevated LDH, elevated CRP, and elevated ESR [41]. The most consistent laboratory finding observed is lymphopenia in adults and raised procalcitonin in children. Urine assays have revealed hematuria, proteinuria, and leukocyturia [42].

Chest X-ray and Computerized Tomography (CT) scans are a sensitive routine imaging tool for COVID-19. Most standard patterns observed on chest CT were ground-glass opacity, ill-defined margins, smooth or irregular interlobular septal thickening, air bronchogram, crazy-paving pattern, and thickening of the adjacent pleura [43].

*Management of COVID-19:* Management of COVID-19 cases is dependent on the triage approach based on the severity of clinical manifestations. Observation, isolation, and quarantine form the basis for managing mild cases. Moderately ill patients are treated with high-flow nasal cannula along with antiviral therapy. Patient isolation and close observation, including monitoring oxygen saturation, are essential in every case. If the patient develops refractory hypoxemia, then administration of Nitric oxide inhalation, neuromuscular blockers, and endotracheal intubation is initiated. Mechanical ventilation using extracorporeal membrane oxygenation is needed in severe cases [44],[45].

Complicated COVID-19 patients can present with shock, renal failure, cardiac failure, and pulmonary edema. In the presence of shock with acute renal failure, the negative fluid balance needs to be achieved by dialysis. Antimicrobials and antifungals are used for pre- and post-exposure prophylaxis. This prevents illness from SARS-CoV-2 and also reduces the risk of acquiring secondary infection. Fluid management is important to reduce pulmonary edema. Glucocorticoids are best avoided due to their harmful effects in viral pneumonia and ARDS. Rescue therapy by administration of intravenous infusion of vitamin C has been suggested to attenuate vascular injury and systemic inflammation in sepsis and ARDS [46]–[49].

*Role of Caribbean Public Health Agency:* Caribbean Public Health Agency (CARPHA) was established legally in July 2011 by Caribbean community member states. It began its operation in January 2013. It issues regional response to public health institutions during natural disasters, surveillance and management of non-communicable and communicable diseases, and contributes to global health agreements in compliance with international health regulations. Once WHO announced COVID-19, a pandemic, CARPHA upgraded the disease transmission risk as “very high” based on international risk assessment guidelines and initiated the following actions: 1) coordinating regional preparedness and activating incident management team, 2) issued situation reports to its members states, 3) developed travelers' air and seaport guidelines, 4) released updated case definitions, statistical and preventive strategies to the health authorities, and 5) security cluster tracing of passengers from high-risk countries [2],[25].

*Vaccination:* The CARPHA- CRS (Caribbean Regulatory System) collaborates with the WHO and the Pan American Health Organization (PAHO) to recognize regulatory authorities used in its procedure for the verification of COVID-19 products that have been assessed by trusted authorities and recommended for emergency use authorization [28]. Information technology is also an important part of the vaccination drive. It creates awareness among the people, providing an important technical framework to better respond to challenges arising to the global health system [28]. As per the information by CARPHA on Jun 28, 2021, 14 Caribbean countries have received the COVID-19 vaccine from the COVAX facility. COVAX is co-led by the Coalition for Epidemic Preparedness Innovations (CEPI), Vaccine Alliance, and WHO, with UNICEF as a key delivery partner and PAHO as the procurement agent in the Americas. This facility aims to ensure fairer global vaccine distribution [50] and address the prevailing inequities [51], but the end result can only be assessed after some time based on fulfilling its commitments to the nations [52].

Based on data collected by the Global Change Data Labs from Our World in Data site, between 2.6% and 66% of persons in the CARPHA Member States have been fully vaccinated against COVID-19. Based on the PAHO Dashboard of COVID-19 Vaccination in the Americas as of Jun 25, 2021, among the CARPHA Member States, vaccine administration (at least one dose) ranges from 8.4 to 142 doses per capita. Approximately 2,211,162 doses have been administered, with an average of 62 doses per capita among CMS (Table 1) [2].

**Table 1.** Vaccination coverage in the CARPHA member states.

Country	Vaccine(s)	Number of Doses	Doses per 100 persons
Anguilla	AstraZeneca — Vaxzevria	15,789	85.7
Antigua & Barbuda	AstraZeneca — not specified	63,755	64.6
Aruba	Information not available	125,182	116.8
Bahamas	AstraZeneca — not specified	79,246	20
Barbados	AstraZeneca — not specified	161,059	56
Bonaire	Pfizer BioNTech — Comirnaty	26,941	134
Belize	AstraZeneca — not specified	106,010	26.2
Bermuda	Information not available	78,413	108.9
Cayman Islands	Pfizer BioNTech — Comirnaty	89,619	142
Curacao	Pfizer BioNTech — Comirnaty	165,545	100.5
Dominica	Beijing CNBG — Inactivated; AstraZeneca — not specified	39,309	52.9
Grenada	AstraZeneca — not specified	31,494	27.9
Guyana	Information not available	335,091	42.4
Jamaica	AstraZeneca — not specified	249,938	8.4
Montserrat	AstraZeneca — Vaxzevria	2,640	48.9
Saba	Information not available	1,300	67.3
Sint Eustatius	Information not available	2,348	74.8
Sint Maarten	Pfizer BioNTech — Comirnaty	38,290	86.2
St. Kitts & Nevis	AstraZeneca — not specified	35,659	65.8
St. Lucia	AstraZeneca — not specified	51,849	28.1

*Vaccine hesitancy:* Vaccination is an effective way to curtail the burden of COVID-19, in which success depends on a high acceptance of the vaccine. A study by Syed et al. in their survey on vaccine acceptance indicated a high acceptance rate of the COVID-19 vaccine among Malaysians [53]. A French study among the working age population found that vaccine hesitancy was lower when herd immunity benefits were communicated; and in working versus non-working individuals. The study further noted that predicted hesitancy was highest for vaccines made in China with 50% efficacy and a 1 in 10,000 risk of serious side effects (vaccine acceptance 27.4% [26.8–28.0]), and lowest for a vaccine made in the EU with 90% efficacy and a 1 in 100,000 risk of serious side effects (vaccine acceptance 61.3% [60.5–62.1]) [54]. A Libyan study by Elhadi et al. reported that regarding vaccine acceptance and efficacy, 12,006 (79.6%) reported their willingness to take the vaccine with an efficacy of 90% or more, 9143 (60.6%) with an efficacy of 70% or more, and only 6212 (41.2%) with an efficacy of 50% [55]. Another study from Australia conducted by Seale and colleagues found encouraging results about the public perceptions and behaviors where 80% (n = 1143) agreed with the statement that getting vaccinated for COVID-19 would be a good way to protect against COVID-19 infection [56]. As emphasized by Harrison et al., public confidence in vaccination programs depends on the work they do for the community — social, political, moral, and biological. The concept of public health and its programs must be broader than the delivery of the vaccine technology itself [57].

*Surveillance:* Active surveillance to COVID-19 has been the mainstay for disease detection and contain transmission. All epidemiological investigations and disease outbreak containment/restriction measures were implemented under the national infectious disease Act [58]. Multipronged surveillance strategies adoption includes case definition, contact tracing of COVID-19 confirmed patients, and active surveillance among high-risk patients (with pneumonia, ICU patients). COVID-19 transmission containment measures are case isolation, quarantine of suspect and probable cases, active monitoring of contacts, country-wise border and travel controls, and community education regarding preventive measures [59]. Surveillance in hospitals includes testing for COVID-19 in the following groups: 1) Hospitalized patients with pneumonia 2) ICU patients with infective etiology 3) Patients presenting with flu-like illness at OPD's (government hospitals/private care clinics) 4) Deaths from infectious causes [60].

Surveillance for travelers is mainly aimed to reduce chain transmissions from imported cases. Border control measures include thermal scans, temperature screening (exit and entry screening) for all travelers, denial of travelers entering high-risk countries, and mandatory 14-day self-quarantine [61]. Surveillance for closed contacts includes 14-day quarantine at designated government quarantine centers or homes, monitoring health status daily, video-call quarantined individuals, and phone surveillance to monitor and verify their location [2]. Confirmed COVID-19 patients need to be interviewed to obtain detailed demographics, activity patterns, and clinical manifestation. These details should be from the pre-symptom onset till hospital isolation. This mainly gives access to tracing close contacts (defined as individuals who have spent prolonged time and are within 2 m of a confirmed COVID-19 patient), secondary transmission, and cluster transmission [59],[61].

*Preventive measures:* Many countries in the Caribbean region declared public health emergencies and imposed stringent public health measures in their respective countries [62]. Closure of schools, universities, cultural centers, social, recreational centers, dine-in restaurants, bars, and sporting centers was announced immediately [63]. Stay at home was ordered initially, and this was also followed by a state of emergency and curfew imposed in some Caribbean islands. Closure of non-essential business and adjusted opening hours to grocery stores, supermarkets, and pharmacies were formalized. Public gatherings in funerals were forbidden. Closure of borders to air travel and cruises were announced [64]. A study by Mohsen et al. suggested that media coverage can be considered as an effective way to mitigate the COVID-19 spreading [65].

As it is a highly contagious virus, effective precautions were implemented. The public health officials, regional health authorities, hospitals, local health departments, and community health service personnel have worked diligently with the universities, workplaces, and public to educate and provide frequent updates and resources to restrict the spread of COVID-19. Detailed public health measures included close follow-up of discharged patients following successful treatment, and any suspected, probable cases were screened and notified [66].

Due to effective transmission of the COVID-19 virus from person to person, the following preventive strategies were recommended to the public: Regular and thorough cleaning of hands, preferably with alcohol-based hand sanitizers. Maintaining adequate social distancing, avoiding touching faces, practicing good respiratory and oral hygiene, elderly and patients with comorbid conditions were reported to have a higher risk of death and were thus advised to stay away from public places and prefer homestay. Online/e-learning strategies are being implemented in schools and universities. The face masks were strictly imposed on persons venturing out in public [67]. Further, efficient healthcare sector and environmental sustainability minimize case fatality rates of COVID-19 [68].

## 5. Conclusions

COVID-19 disease transmission can be contained only by effective control of person-to-person transmission, patient isolation and confinement, social distancing, and preventive community containment measures. Successful disease isolation depends on early case detection (before the onset of peak viral shedding). A patient with fever or respiratory illness symptoms and a contact or travel history is considered a highly sensitive case for SARS [2].

Quarantine includes restriction of movement combined with medical observation of close contacts of infected patients during the incubation period. Quarantine is usually done at home or in designated places such as hotels or set up camps. Quarantined contacts record their temperatures and are observed by the public health care teams daily and are further investigated if they develop symptoms. The main reason for quarantine is to avoid human-to-human transmission, which forms the vital link of disease spread [2],[25]. Community containment measures are designed to reduce personal interactions within a community, region, or city. Setting up isolation rooms for staff with strict use of personal protective equipment (PPE, which includes gloves, gowns, eye protection, and N95 respirators), restricting visitor entry, negative pressure rooms, infection control precautions, and a separate triage facility were all recommended and implemented as hospital containment measures. [2],[64].

The psychosocial impact of the COVID-19 pandemic in the Caribbean has been inclined due to prolonged isolation, quarantine, and lockdown periods across the region. Health care professions are at a high risk of anxiety, depression, and distress due to prolonged work exhaustion, lack of personal protective equipment, concern about infecting their families, and witness to patients' death. Corrective guidance, the family's safety, effective disease prevention measures, and positive attitude from their working colleagues can improve the psychological outcome during this pandemic. They have impacted public needs easy access to clinical services, regulatory implementation of infection control measures, and establishment of counseling service centers. Providing psychological first aid is crucial for those who are affected and bereaved family members [69],[70]. Thus far, a limited number of cases have reported intrauterine and perinatal infection caused by vertical transmission in women who developed COVID-19 in late pregnancy. Infants born to COVID-19 suspected or confirmed mothers should be isolated, observed, and evaluated [71]. Confirmed COVID-19 mothers should be cautious during breastfeeding to avoid droplet transmission and contact transmission to the baby. However, no virus has been reported in the maternal milk of COVID-19 positive mothers [72]. Public health measures were implemented early across the region and mainly followed the WHO's guidelines and strategies, restricting the drastic outcome of this zoonotic disease. Data providing real-time disease prevalence, statistical evaluation of high risk, local description on age, and country-wise detailed clinical statistics would improve the disease containment, identify the high-risk population, and reduce the CFR in certain Caribbean countries. Strong regional collaboration between the Caribbean member states is necessary to provide optimal real-time data to the public and implement effective guidance in their respective countries. The role of CARPHA and government health services in restricting disease transmission across the region has been commendable. Many island countries have now started to ease the restrictions in a phased manner.
